# Modeling forecast errors for microgrid operation using Gaussian process regression

**DOI:** 10.1038/s41598-024-52224-y

**Published:** 2024-01-25

**Authors:** Yeuntae Yoo, Seungmin Jung

**Affiliations:** 1 Department of Electrical Engineering, Myongji University, Yongin, 17058 Republic of Korea; 2 Department of Electrical Engineering, Hanbat National University, Daejeon, 34158 Republic of Korea

**Keywords:** Energy grids and networks, Power distribution, Energy grids and networks, Power distribution

## Abstract

Microgrids, denoting small-scale and self-sustaining grids, constitute a pivotal component in future power systems with a high penetration of renewable generators. The inherent uncertainty tied to renewable power generation, typified by photovoltaic and wind turbine systems, necessitates counterbalancing mechanisms. These mechanisms encompass Energy storage systems or conventional thermal fossil-fuel generators imbued with heightened flexibility. Addressing the uncertainty stemming from renewable generators mandates a cost-effective assessment and operational strategy for said compensatory devices. To this end, myriad uncertainty factors warrant scrutiny, conceivably concretized into a unified probability distribution function (PDF) that takes into account their temporal inter-dependencies. Diverse uncertainty factors, characterized by varying marginal distributions and scales, can be assimilated into a multivariate probability distribution through a conversion to normal distributions via rank correlation. However, with the escalation in the number of uncertainty factors embraced within a microgrid context, the endeavour becomes notably intricate when aiming to define conditional probability distributions originating from joint PDFs. This paper presents a method proposing the modelling of net-load forecast error distribution, considering the interplay among uncertainty factors. The approach introduces a data-driven Gaussian process regression technique for training and validating conditional PDFs among these uncertainty factors. Notably, this approach facilitates the transformation of said factors into normal distributions while preserving their inherent marginal characteristics. The resultant conditional density function, as per the proposed methodology, exhibits enhanced suitability for estimating net-load error distribution. Consequently, the conditional density function stemming from this proposed approach demonstrates superior aptitude in approximating the distribution of net load error.

## Introduction

The proliferation of Distributed Energy Resources (DERs), including photovoltaic generators (PV) and wind turbines, is driving a rapid transformation in power system operations. This shift is particularly notable in the escalating adoption of microgrids, which have garnered significant attention for their capacity to serve as localized network operators. Microgrids play a pivotal role in efficiently managing load demand and power generation from DERs within their designated local networks.

Anticipating the trajectory ahead, it’s becoming increasingly apparent that the future of power system control will involve hierarchical structures operating through numerous clusters of microgrids, each integrated with its own set of DERs. This multi-tiered approach to control and coordination presents an intriguing framework for managing the complex interplay between distributed energy sources and the broader power system. As DERs and microgrids continue to shape the landscape of power generation and consumption, refining hierarchical control strategies is poised to play a pivotal role in ensuring the stability, flexibility, and sustainability of modern power systems.

Within a microgrid, the fundamental challenge lies in achieving a harmonious equilibrium between load demand and the power generated by Distributed Energy Resources (DERs), given the restricted pool of controllable power sources. As the integration of DERs intensifies within microgrids, so does the level of uncertainty, necessitating the implementation of supplementary balancing mechanisms for ensuring efficient microgrid operation. To this end, Energy Storage Systems (ESS) emerge as a prominent choice for acting as the primary balancing device in such grids. ESS offers the advantages of swift response times and a versatile operational range, making them particularly suited for handling the dynamic nature of microgrid dynamics.

Nevertheless, the deployment of ESS comes with a financial cost, prompting a significant body of research to focus on optimizing their allocation and assessing their capacity within microgrid settings. These studies strive to identify the optimal positioning of ESS units and determine their appropriate capacity levels, enabling microgrids to effectively manage the uncertainties introduced by variable DER outputs. By achieving an optimal balance between DERs and ESS, microgrids can enhance their resilience, reliability, and overall operational efficiency, further contributing to the evolution of sustainable and responsive energy systems.

The optimization of microgrid operation can be strategically devised to minimize the requisite capacity of Energy Storage Systems (ESS) or alternative balancing mechanisms. To accomplish this, a predictive model utilizes forecasts of Distributed Energy Resources (DERs) generation and load demand within the framework of microgrid optimization. Notably, the precision of these forecasted data significantly influences the assessment of balancing device requirements.

Several methodologies exist for forecasting the inherent uncertainty associated with DERs generation and load demand. Time series analysis techniques, such as Autoregressive Integrated Moving Average (ARIMA) and Seasonal ARIMA (SARIMA), represent straightforward yet effective linear regression models employed for short-term point forecasting^1,2^. More contemporary research integrates machine learning algorithms, with non-linear artificial neural network (ANN) models emerging as a robust approach for short-term point forecasting of load demand^3^.

Machine learning-based methods offer distinctive advantages by accommodating supplementary parameters for forecasting^4^. This adaptability enables these approaches to capture complex relationships in a sophisticated manner, culminating in a heightened accuracy when forecasting data^5^. An optimal Long Short-Term Memory (LSTM)—Recurrent Neural Network (RNN) based model can be developed for 30-min and 24-h ahead electrical load forecasting^6^ These models can be further improved through additional heuristic analysis of their network configuration. By tapping into machine learning’s capabilities, microgrid optimization can harness more comprehensive information, enabling a more accurate determination of the balancing devices needed to ensure the reliable and cost-effective operation of the microgrid.

With the ever-widening integration of Distributed Energy Resources (DERs), the comprehension and utilization of uncertainty distribution information are assuming a paramount role in both power system operation and planning. Recent developments have led to a growing focus on quantile forecasting as a potent tool for evaluating the distribution of uncertainty. This approach holds significance as it directly provides insights into the distribution characteristics in a non-parametric manner^7^.

An especially noteworthy advancement in this realm is the emergence of neural network-based quantile regression, as introduced in^8^. This methodology endeavours to construct a comprehensive conditional density curve for future load, thereby offering a comprehensive depiction of the load distribution characteristics. Furthermore, Gaussian process quantile regression^9^ has also gained attention, demonstrating its capacity to effectively capture the distribution uncertainties inherent in power system forecasting. In an endeavor to achieve even more accurate conditional density forecasts, recent studies have explored approaches such as ensemble techniques that amalgamate multiple methods^10^. These approaches have proven to be noteworthy, as the combination of clustering methods and probabilistic load forecasting can potentially reduce load forecasting errors in a microgrid and facilitate the analysis of the relationship between forecasting accuracy and load characteristics^11^.

By leveraging these innovative techniques, power system operators and planners stand to glean more nuanced insights into the uncertainties surrounding load demand and other relevant parameters, thereby facilitating more robust decision-making processes in the face of dynamic and evolving power system dynamics.

Indeed, a substantial portion of research in the domain of probabilistic forecast methods has predominantly concentrated on load forecasting. In this context, several studies have delved into the analysis of conditional forecast errors by considering external factors, such as temperature and time, which exert a direct influence on the accuracy of predictions. However, in the context of microgrid operation, the concept of net load introduces a multifaceted dynamic.

The net load in a microgrid emerges as a synthesis of various uncertainties associated with forecasts for PV and wind generation, coupled with load forecast data. This amalgamation of uncertainties gives rise to intricate and nuanced conditional dependencies that interlink the net load with each individual uncertainty factor. The complexity of these inter-dependencies demands a comprehensive and intricate approach to assessing and managing the net load in microgrid operation.

In light of these intricacies, developing advanced probabilistic forecast methods that consider the intricate conditional dependence among these multiple sources of uncertainty becomes crucial. This approach can provide a more accurate and comprehensive understanding of the potential range of net load scenarios, ultimately enhancing the resilience and adaptability of microgrid operations in the face of diverse and dynamic uncertainty sources.

The interplay of correlation among uncertainties and the accurate estimation of forecast errors holds a pivotal role in microgrid operation^12^. However, within the existing body of literature, there appears to be a dearth of comprehensive discussions that depict net load as a joint probability density function shaped by the convergence of various uncertainty factors. Zhang et al. have taken steps towards addressing this challenge by introducing a parametric estimation approach that encompasses uncertainties related to PV, wind, and load forecasts. Additionally, they calculated rank correlation to inform generator dispatch decisions^13^. Building upon this foundation^14^, proposed a method wherein an adaptive correlation coefficient matrix, evolving over time, was introduced. This matrix served as a means to model the correlated uncertainties arising from variables such as wind speed and the electric price elasticity of customers. The use of parametric estimation facilitated a more sophisticated and adaptive portrayal of these inter-dependencies. Furthremore, EV-charging-load-addressable MG capacity planning optimisation approach is proposed by characterising various sources of parametric uncertainty in an integrated manner to appropriately reflect the underlying correlations of the uncertainty factors, thereby preserving the associated multivariate relationships^15^. Until now, the uncertainty of components in microgrid planning and operation has been seldom taken into account. Furthermore, only a limited number of papers have explored the correlations between uncertainties and their corresponding forecast values.

In this paper, we present a novel approach using non-parametric Gaussian Process Regression (GPR) to estimate the conditional net load forecast error within a microgrid system, considering the influence of other uncertainties. To achieve this, we construct forecast models for load, photovoltaic (PV) generation, and wind generation. These models leverage autoregressive recurrent neural networks with exogenous inputs and are supplemented with data from Numerical Weather Prediction (NWP) systems sourced from the Global Data Assimilation and Prediction System (GDAPS) (Supplementary Information 1).

The proposed methodology operates as follows: leveraging the forecast values of uncertainties related to load, PV generation, and wind generation, we apply GPR to determine the conditional density distribution of net load error. This approach enables us to capture the intricate inter-dependencies between different sources of uncertainty and their cumulative effect on netload forecasts. To validate the effectiveness of our approach, we verify the derived conditional density distribution against actual net load data. Contributions of this paper are as follows:

- Proposed a method to build conditional density of net load forecast error in terms of various uncertainty factors in a microgrid, and
- Combined multiple uncertainty factors based on a non-parametric quantile Gaussian regression process to increase PDF estimation accuracy of net load forecast error.

The structure of the paper is outlined as follows: section “Net load forecast model with NWP data” outlines the development of forecast models for individual uncertainty elements within a microgrid, which are then aggregated to formulate net load forecasts. Moving on to section “Net load forecast error modelling using Gaussian process regression”, the process of generating Probability Density Functions (PDFs) for each uncertainty is elucidated, involving kernel-based estimation and transformation to a normal distribution. Furthermore, section “Net load forecast error modelling using Gaussian process regression” encompasses the formulation of the conditional PDF for net load forecast error through Gaussian regression processes. The efficacy of these methodologies is subsequently demonstrated through case studies in section “Case studies”.

## Net load forecast model with NWP data

PV generation is closely linked to the amount of solar irradiation received at ground level. The efficiency of a PV generator is influenced by factors like the tilt angle and azimuth of the PV panel. Given the complexity of knowing the specifications of every individual PV installation, it’s pragmatic to consider a standardized PV generator with uniform parameters. Consequently, forecasting PV generation in a specific area is achieved through predicting solar irradiation at ground level corresponding to the site in question. This approach simplifies the forecasting process by focusing on solar irradiation, a crucial determinant of PV performance, without the need for exhaustive details about each unique PV setup. The output of the equivalent PV generator can be approximated by

1 $$\begin{aligned} P_{PV} = C_{PV}\frac{I_{PV}}{I_{0}}[1-\mu (T_{PV}-T_{0})], \end{aligned}$$

where $C_{PV}$ is the capacity of PV generator, $I_{PV}$ is the solar irradiation level in $W/m^{2}$, $T_{PV}$ is the temperature during the operation, $\mu$ is the constant temperature parameter of the PV panel, and $I_{0}$ and $T_{0}$ are standard irradiation and temperature constants under Standard Test Conditions (STC), respectively^16^.

Predicting the quantity of solar irradiation at ground level is complicated due to variables such as cloud cover and atmospheric moisture. Conversely, the extraterrestrial solar irradiation approximated outside Earth’s atmosphere, can be characterized by the relative movement of the sun as observed from our planet^17^. The level of extraterrestrial solar irradiation can be calculated by

2 $$\begin{aligned} G_{0} = G_{SC}\left( 1+0.033\cos {\frac{360n}{365}} \right) \times \left( \cos {\phi }\cos {\rho }\cos {\omega }+\sin {\phi }\sin {\rho } \right) , \end{aligned}$$

where $G_{SC}$ is the solar coefficient, *n* is the number of days of each year starting from January 1st, $\phi$ is the latitude, and $\rho$ is the declination. $\omega$ is the hour angle defined by

3 $$\begin{aligned} \omega = 15*(t-12) + (\lambda -120), \end{aligned}$$

where *t* is the hour of the day and $\lambda$ is the longitude value. The solar declination angle $\rho$ for a given day of the year can be approximated by

4 $$\begin{aligned} \rho = 23.5*\cos {\left( 360\times \frac{n-172}{365} \right) }, \end{aligned}$$

The extraterrestrial solar irradiation, denoted as $G_{0}$, serves as a foundation for estimating ground-level solar irradiation alongside cloud cover information. Cloud cover is quantified on a scale of 0 to 10, where 0 signifies an absence of clouds and 10 denotes complete cloud coverage. The correlation between measured ground irradiation, extraterrestrial irradiation, and cloud cover has been established through regression analysis^18^. More recent research has showcased improved accuracy through the utilization of Artificial Neural Network (ANN)-based regression^19^.

Considering the predictability of hourly PV generation profiles with time, a viable model for forecasting PV generation power is an autoregressive neural network model. This model incorporates exogenous input parameters such as cloud cover and extraterrestrial solar irradiation data. This approach aligns well with established practices in estimating the hourly PV generation profile^20,21^. By integrating these parameters into the model, the prediction accuracy of PV generation power can be substantially enhanced, facilitating more reliable energy generation forecasts.

**Figure 1:**
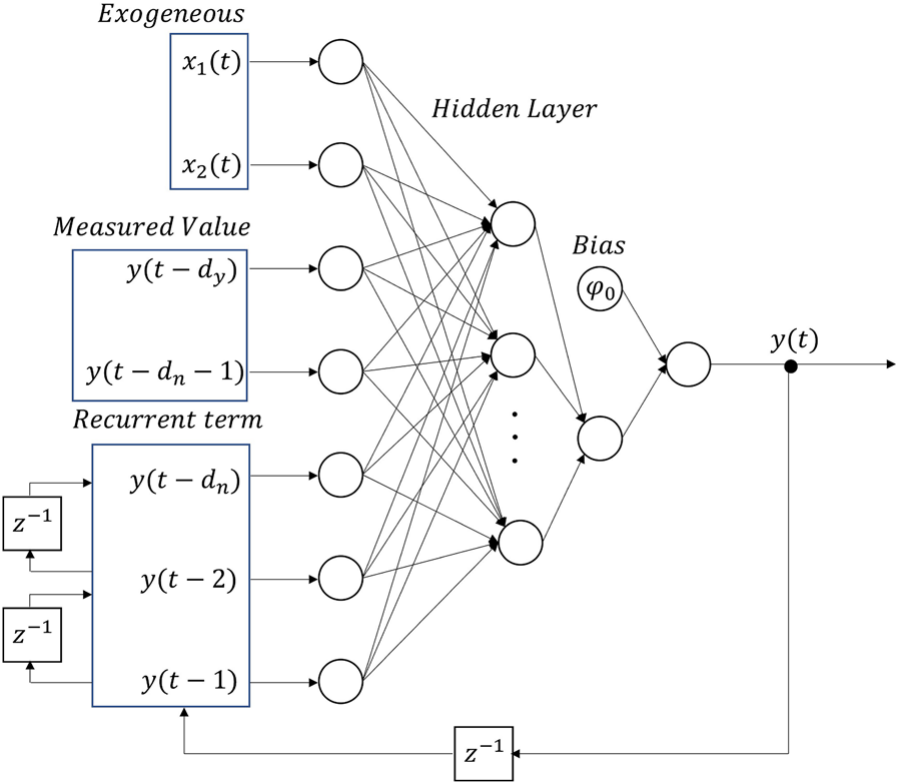
Autoregressive neural network with exogenous inputs for uncertainty forecasting.

In this study, the models for forecasting photovoltaic (PV) generation and load are founded upon an autoregressive neural network model with exogenous inputs. The schematic representation of this framework is presented in Fig. 1.

In the context of PV generation forecasting, the ARNN based model incorporates both cloud cover data, projected through the Numerical Weather Prediction (NWP) forecast model, and extraterrestrial solar irradiation as exogenous inputs. The autoregressive input component encompasses both recurrent term and field measurement data. To determine the balance between the recurrent term and field-measured values, this ratio is modulated based on the forecasting horizon’s duration. With 48 delay steps, corresponding to each hourly interval across the past 48 h, the autoregressive input is designed. Conversely, the exogenous inputs are deterministic variables and thus do not involve any delays.

**Figure 2:**
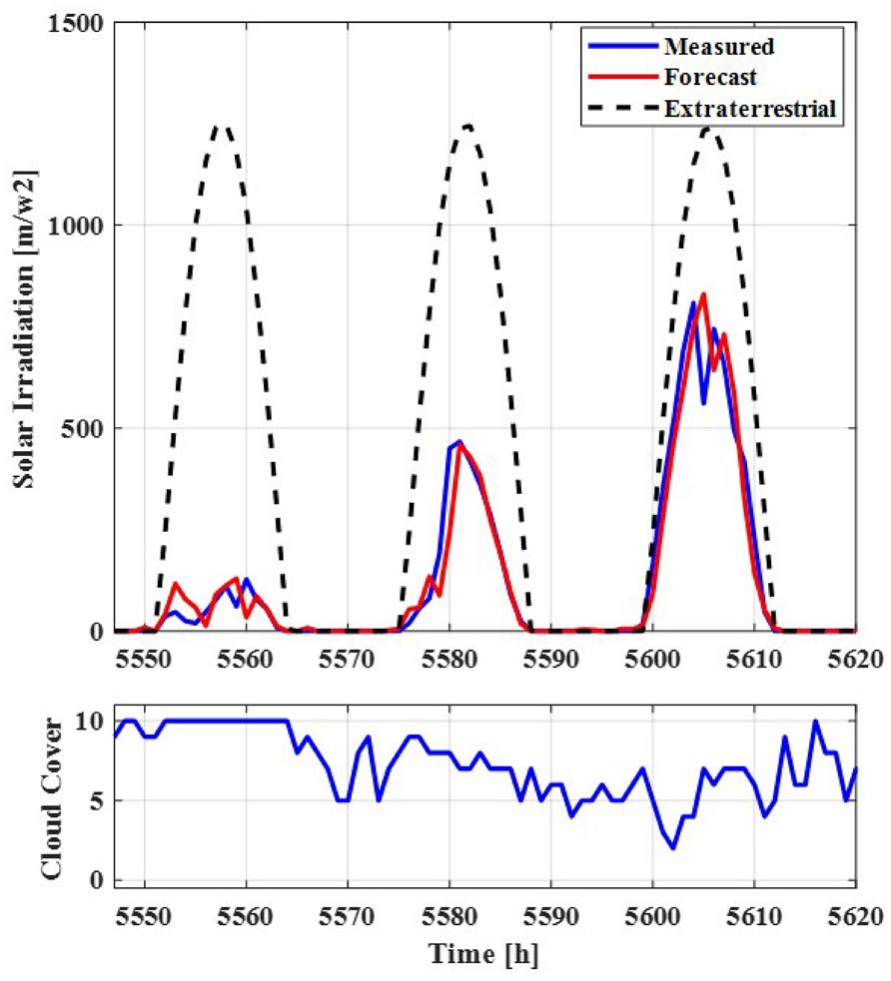
Measurement data of solar irradiation and its forecasting data based on autoregressive neural network with extraterrestrial irradiation value and cloud cover data.

In Fig. 2, the upper figure exhibits the extraterrestrial irradiation value (depicted by the black-dotted line) and cloud cover data (represented by the blue solid line) collected from January 2018 to December 2018 at a meteorological station located in Gosan, Jeju Island, Korea (26.284′′ N, 53.622′′ E, 71.4 m above sea level)^22^. Moving to the lower figure, the blue solid line corresponds to measurement data of solar irradiation, while the red solid line illustrates forecasted solar irradiation data. The forecast data is generated using an autoregressive network incorporating extraterrestrial irradiation value and cloud cover data.

The extraterrestrial irradiation data effectively captures variations in sunrise and sunset timings across different seasons, as well as changes in solar intensity. It’s evident that cloud cover data exhibits a notable correlation with ground-level solar irradiation data, except during instances of partial cloud cover. However, the utilization of autoregressive inputs has the potential to mitigate forecast errors that arise in such partially cloudy conditions. This observation underscores the ability of the autoregressive model to enhance forecast accuracy, even in situations characterized by fluctuating cloud cover.

In the realm of load demand prediction, there exists robust serial correlation for consecutive lags. This characteristic allows for the effective utilization of empirical load demand data from previous days or weeks to enhance estimation accuracy^2^. By merging a conventional time series framework with neural network techniques, load demand forecasting can achieve greater precision^23^.

The load demand forecast model employed in this study follows the structure presented in Fig. 1. It incorporates temperature data and a categorical variable to distinguish holidays. The autoregressive inputs consist of 168 consecutive delay steps, corresponding to a 7 days period encompassing 168 hourly data points. Temperature data is sourced from the same Gosan station as the solar irradiation data, localized within the microgrid deployment area rather than spanning the entirety of Jeju Island. Conversely, load demand data is obtained from the local Transmission System Operator (TSO) on Jeju Island, consolidated from the entire island. To align with the microgrid context, this load demand value is appropriately scaled down. The load demand forecast model employs five hidden layers to optimize prediction performance.

Predicting wind speed and wind generation power over extended time frames presents a challenge. While wind exhibits greater stability compared to solar irradiation, its intermittent nature makes estimation more complex. Consequently, for short-term wind forecasting, a time series model employing a limited delay is often adopted^1^.

In the context of longer time horizons, forecasting wind generation power necessitates the involvement of an NWP model, which calculates atmospheric interactions on a global scale. The wind forecast model in this study is constructed upon the ARIMA model, complemented by NWP data. The ARIMA model incorporates two autoregressive inputs and a sole moving average delay, optimizing its predictive capability for wind generation power forecasts spanning extended periods. The combination of NWP data and ARIMA modelling provides a comprehensive approach to addressing the complexities associated with forecasting wind generation over longer horizons.

**Figure 3:**
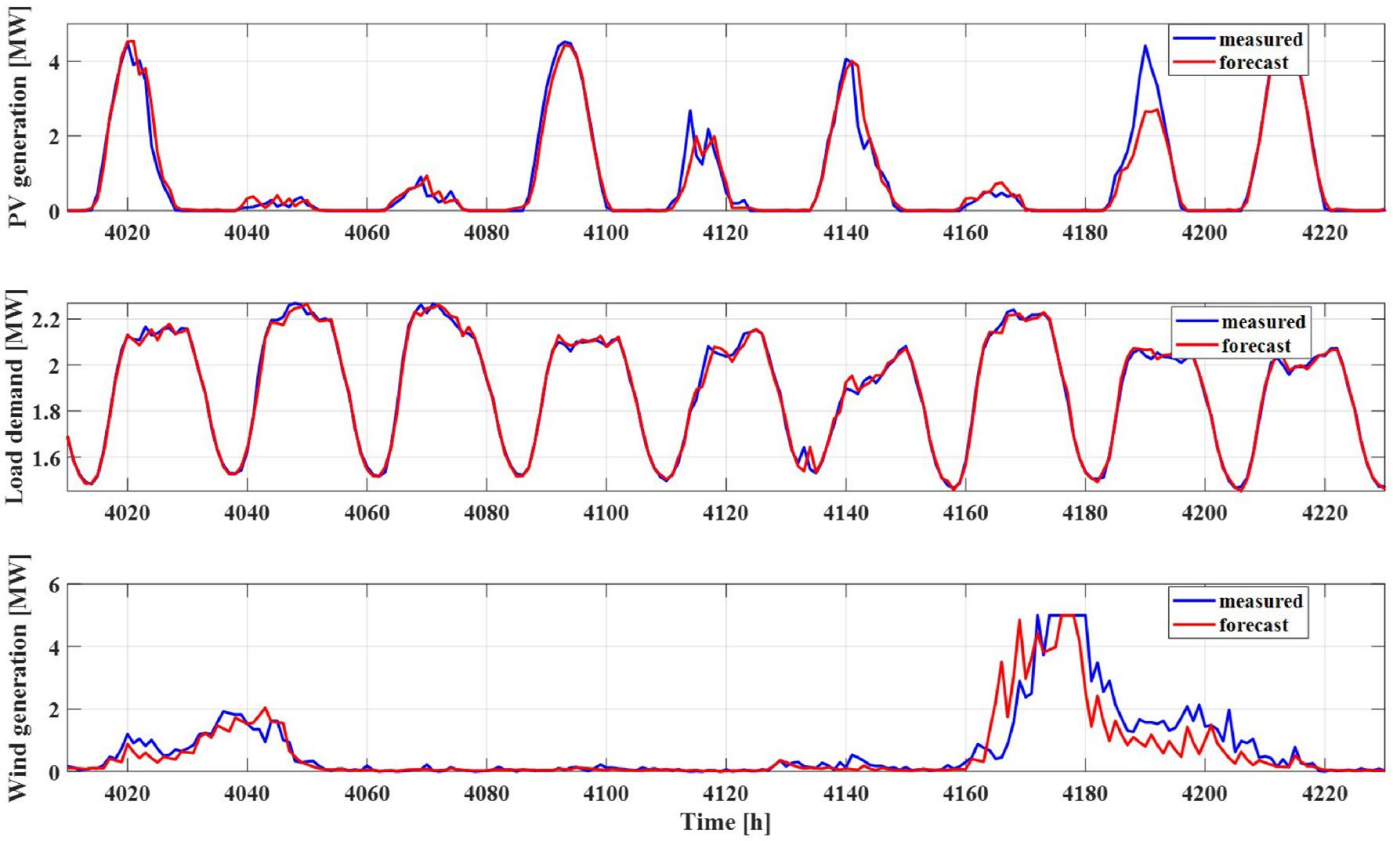
Comparison between field measurement data and their corresponding forecast values.

In Fig. 3, a comparative analysis is presented, contrasting measurement data with forecast data for PV generation power, load demand, and wind generation power. It’s noteworthy that point forecasts for PV generation power display reduced accuracy during instances of partial cloud cover. This decrease in accuracy is attributed to the fact that the NWP forecast model, designed for wide-scale predictions, is less suitable for estimating PV generation within smaller areas due to its macroscopic nature. Notably, autoregressive inputs serve as a corrective mechanism when forecast errors attain significance, enhancing forecast accuracy.

Comparatively, load demand forecasts exhibit higher accuracy in relation to both PV and wind generation power forecasts. This can be attributed to the inherent attributes of load demand measurement data, characterized by reduced noise and elevated serial correlation.

The precision of wind generation power forecasts trails behind that of the other two components. This lower accuracy arises from the non-periodic and relatively less exogenous nature of wind data available for estimation. While the NWP model contributes to enhancing the accuracy of long-term wind speed forecasts, its spatial and temporal resolution does not meet the demands of short-term horizons or small-scale areas, underscoring the challenges faced in short-term wind forecasting.

**Figure 4:**
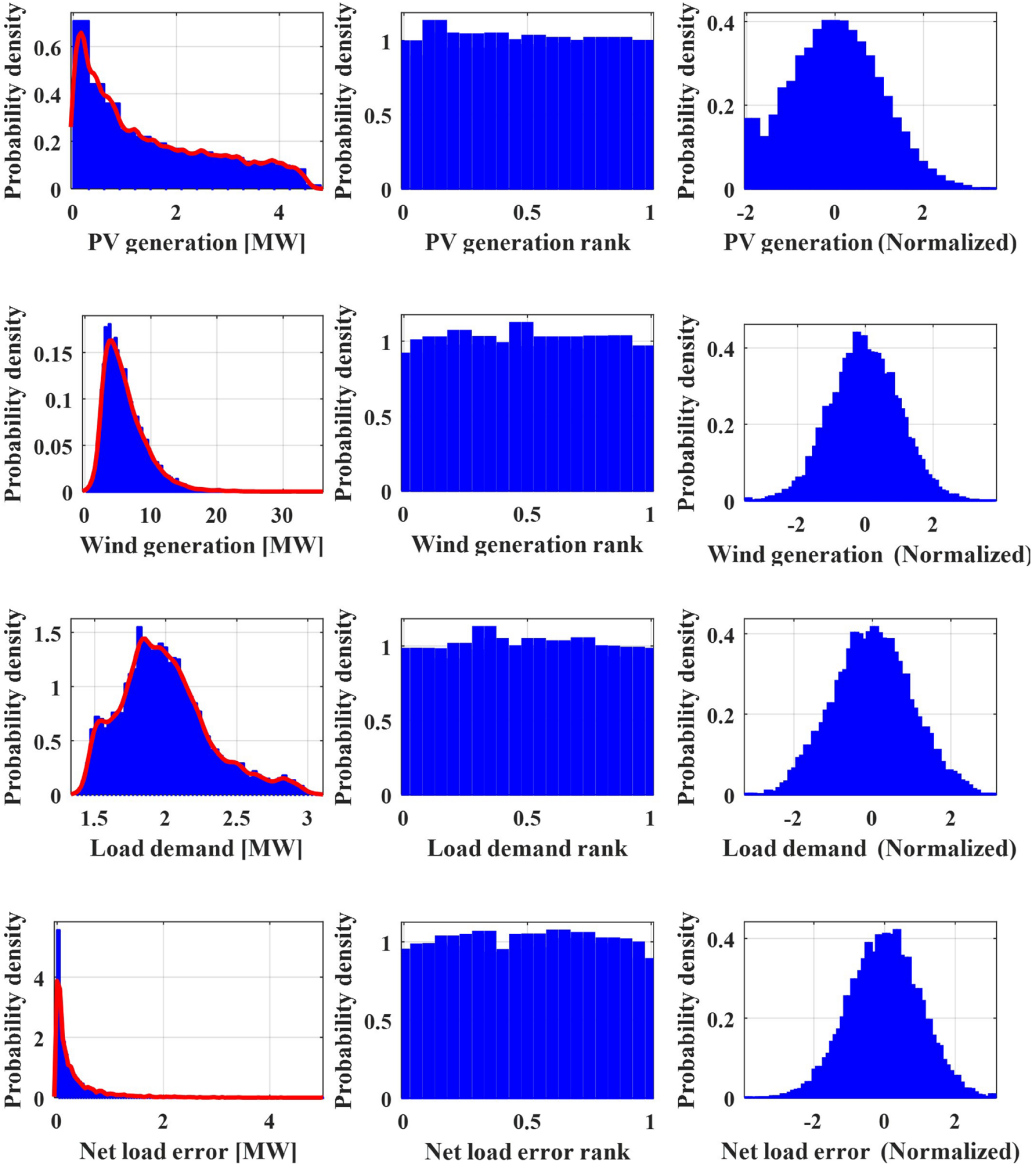
Transformation of the marginal distribution of uncertainties to a normal distribution using KDE.

## Net load forecast error modelling using Gaussian process regression

The net load within our microgrid context is computed by subtracting the combined wind and solar generation power from the total electric demand. In microgrid operation, achieving balance in the net load requires the intervention of balancing devices that can supply or consume active power. Optimal operation costs for a dispatched generator can be attained under the assumption of a perfect operational forecast. However, given the practical imperfection of uncertainty forecasts, managing the operation of balancing devices in the presence of forecast error becomes a crucial consideration. To this end, a robust strategy is required to address the uncertainties arising from these forecasts.

The probability distribution of forecast error for each uncertainty factor, such as PV generation, wind generation, and load demand, can be modelled as a conditional Probability Density Function (PDF) contingent on the point forecast value of that particular factor^24^. For instance, the probability distribution of PV generation forecast error can be constructed as a conditional PDF predicated on the point forecast value of PV generation. However, due to the intricate relationships among uncertainties, directly deriving the probability distribution of net load forecast error from the forecast errors of individual uncertainties is not feasible. Consequently, a method is needed to generate the probability distribution of net load forecast error while considering the underlying uncertainties. This method should encompass the complex interplay among these uncertainties and provide a comprehensive assessment of net load forecast errors.

In the context of statistical analysis, the correlation existing among multiple random variables can be effectively represented through a joint PDF. Typically, a Gaussian joint PDF is well-suited for capturing the correlation among multiple random variables. In the scope of this paper, the three key uncertainty factors-namely, PV generation, wind generation, and load demand-are under consideration. Hence, the objective is to establish a multivariate Gaussian joint PDF that accommodates these three uncertainties as well as net load forecast error. This joint PDF serves as a foundation for deriving the marginal PDF of net load forecast error. Given the distinct value ranges of each uncertainty, the introduction of rank correlation is proposed to appropriately construct this joint PDF.

To establish the probability distributions for each of the random variables, real-world field measurement data from the Gosan meteorological station are employed, as illustrated in the leftmost panel of Fig. 4. By applying Kernel Density Estimation (KDE) to the historical data, the PDF for each random variable (represented as $f_{X_{1}}$ for PV generation, for instance) is estimated. These PDFs are visualized using red solid lines in the leftmost panel of Fig. 4. In the subsequent column of Fig. 4, the CDF transformation yields uniform distributions, providing a foundation for further analysis.

To capture the interdependence of these uncertainties, a multivariate CDF is developed in the form of a copula function. This multivariate CDF subsequently aids in deriving a conditional PDF contingent on the point forecast value of each individual uncertainty factor.

While the copula model is effective in capturing the correlation between multiple random variables, its accuracy tends to diminish when constructing a conditional CDF for a specific random variable, especially when intricate correlations stem from other uncertainty factors. A notable example of this challenge arises with the probability distribution of net load forecast error and the probability distribution of PV forecast during nighttime hours. During these periods, PV forecast values are consistently zero, while net load forecast error varies. This divergence indicates that the conditional PDF for net load forecast error must be formulated with a consideration of the nonlinear characteristics exhibited by each uncertainty factor.

To address this, the approach employed in this paper involves utilizing the GPR model for creating the conditional PDF of net load forecast error based on the point forecast values of each uncertainty factor. To facilitate this modelling, each uncertainty factor’s uniform distribution is transformed into a Gaussian PDF with a mean of zero using its respective inverse CDF. The outcomes of this transformation process are showcased in the rightmost figures of Fig. 4. This approach is designed to account for the complex and nonlinear interactions among the uncertainty factors, resulting in a more accurate depiction of the conditional PDF for net load forecast error.

A Gaussian process refers to a set of random variables in which any finite subset follows a joint Gaussian distribution^25^. In the context of this paper, a Gaussian process is harnessed to establish a Bayesian framework for regression analysis. The Gaussian process defines a probability density over function and can be defined as

5 $$\begin{aligned} f(x)\sim GP(m(x),k(x_l,x_m)), \end{aligned}$$

where $x_l$ and $x_m$ are d-dimensional vectors. *m*(*x*) is a mean function and $k(x_l,x_m)$ is a covariance function, which is also known as a kernel function. The kernel function *k* used for the GPR model is defined by

6 $$\begin{aligned} k(x_{l},x_{m}) = {\sigma _{f}}^2\exp {\left( -\frac{1}{2}\sum _{n=1}^d\frac{(x_{ln}-x_{mn})^2}{\sigma _{n}^2}\right) }, \end{aligned}$$

where $\sigma _{f}$ is the hyper-parameter determining kernel size, $\sigma _{n}$ is the length scale for predictor *n*, and *n* is the predictor index as $n=1,2,\ldots ,d$. In this paper, $d=3$, as there are three predictor variables. We make the assumption that the provided test data and the actual function outputs at the corresponding test points are distributed jointly as a multivariate normal distribution. Hence, the entire data set counts only as a partial observation sampled from the multivariate joint normal distribution as

7 $$\begin{aligned} \begin{bmatrix} y\\ f^{*} \end{bmatrix} \sim N\bigg ( \begin{bmatrix} 0\\ 0 \end{bmatrix}, \begin{bmatrix} K_{X,X} &{} \quad K_{X,X^{*}}\\ K_{X^{*},X} &{} \quad K_{X^{*},X^{*}} \end{bmatrix} \bigg ), \end{aligned}$$

where *y* is an observed output vector, and $f^{*}$ is an unobserved true function output of the test point. *K* is a matrix of all kernel similarities; *X* is input for observed output vector, and $X^{*}$ is test inputs. Since *y* and $f^{*}$ are normally distributed, the posterior distribution over $f^{*}$ is also a multivariate normal distribution as

8 $$\begin{aligned} f^{*}|X^{*} \sim N(\mu _{f^{*}},\Sigma _{f^{*}}), \end{aligned}$$

where the mean vector, $\mu _{f^{*}}$, and covariance, $\Sigma _{f^{*}}$, are given by

9 $$\begin{aligned} \mu _{f^{*}} = K_{X^{*},X}K_{X,X}^{-1}X^{*} \end{aligned}$$

and

10 $$\begin{aligned} \Sigma _{f^{*}} = K_{X^{*},X^{*}}-K_{X^{*},X}K_{X,X}^{-1}X^{*}K_{X,X^{*}}, \end{aligned}$$

respectively.

The GPR model used to estimate the net load forecast error is trained and tested using the probability distribution that follows a normal distribution for each uncertainty factor and net load forecast error.

## Case studies

In this section, we assess the forecasting performance of the GPR model by comparing it with field measurement data. The solar irradiation and wind speed data were gathered from the Gosan meteorological station and employed to create forecast models, as depicted in Fig. 3. Load demand information was obtained from a nearby TSO and scaled down for the purposes of this study. A comprehensive system description is provided in Table 1. The load demand varies between 1379 and 3120 kW, exhibiting similar patterns in a 24 h interval. With a PV generation capacity of up to 5 MW, which is sufficient to meet the load demand during periods of high solar irradiation, the wind generation capacity is identical to that of PV. Both PV and wind generation are assumed to be located in adjacent positions, and thus, the datasets are retrieved from a single meteorological station. Prediction data, based on the dataset from the meteorological station and a combined Numerical Weather Prediction (NWP) model, are provided in 12 h increments.

**Table 1:** Microgrid system description for case study.

System description	
Load	Min 1379 kW $\sim$ Max 3120 kW
PV Generator	STC capacity 5000 kW
Wind Generator	Rated Capacity : 5000 kW wind speed (Min: 2 m/s, Max: 25 m/s, Rated: 15 m/s)
Location for NWP	Latitute 33.29 , Longitude 126.16
Period for Training	Jan.01.2019 $\sim$Dec.30.2019
Operation Period	Jan.08.2020 $\sim$Dec.01.2020

**Figure 5:**
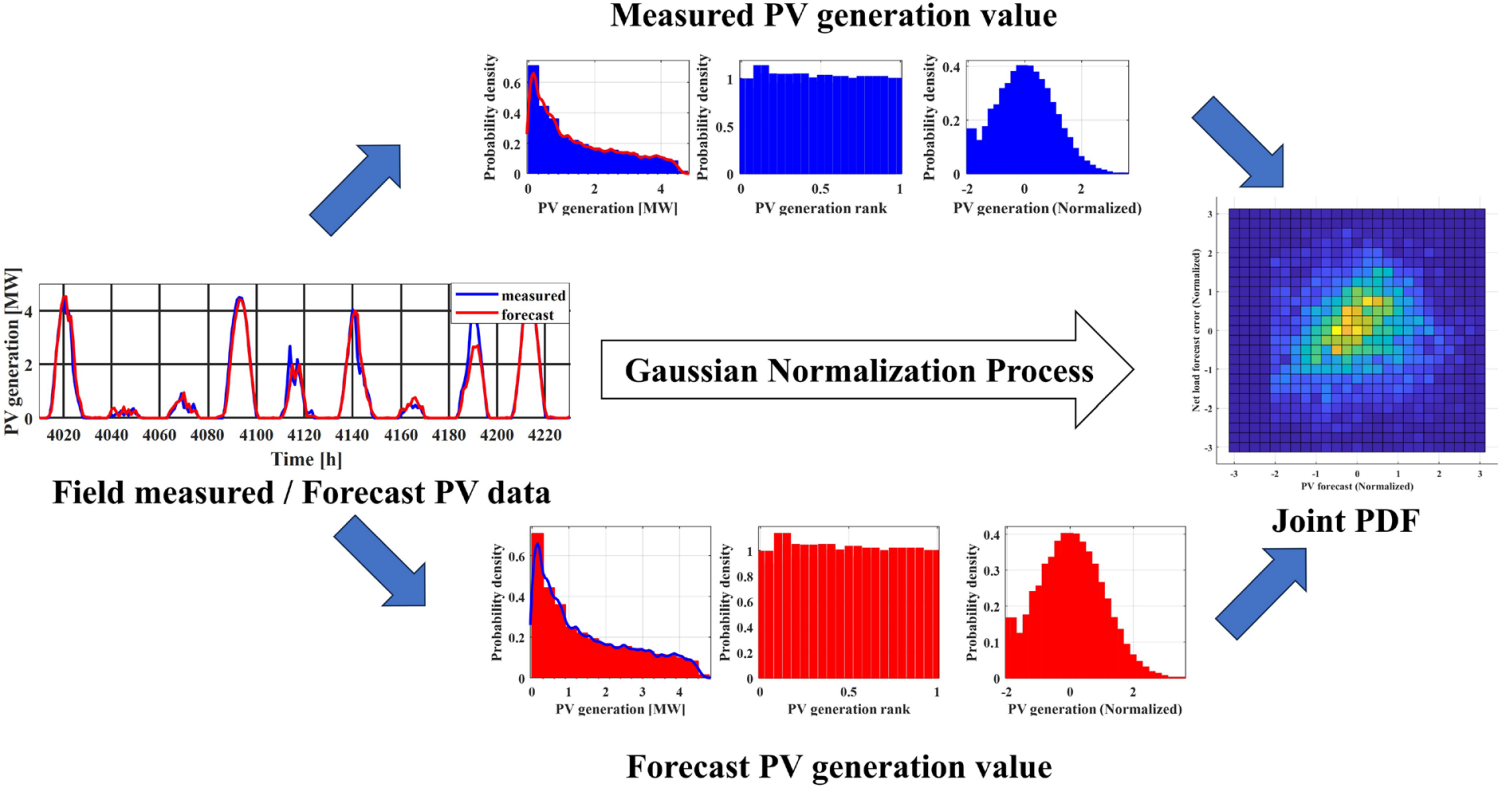
Gaussian normalization process for forecast error joint PDF with measured value.

**Figure 6:**
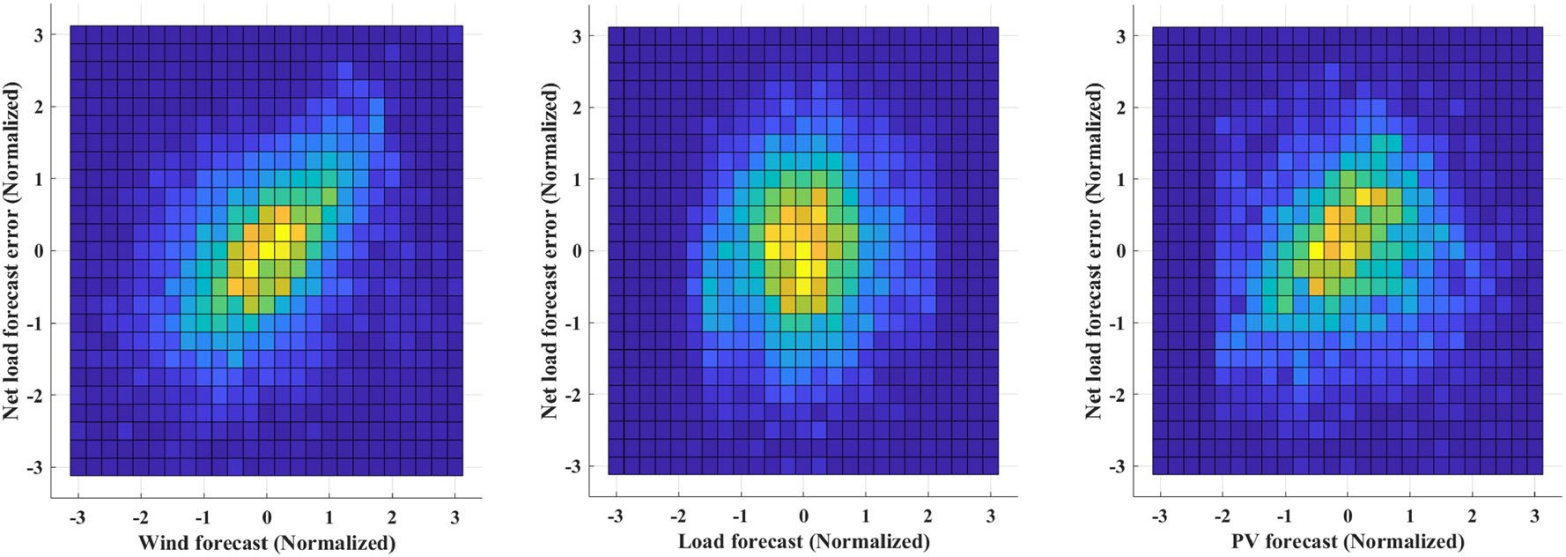
Joint probability density of net load forecast error with respect to each uncertainty forecast.

The collection of empirical data is converted into a normal PDF using KDE and the inverse CDF of the Gaussian PDF. As shwon in Fig. 5, The conversion from the original distribution to a uniform distribution is achieved through the use of PDF and its CDF, which are constructed using KDE processes. The red line in the left-most figure represents the PDF resulting from the KDE process. The uniform distribution displayed in the middle figure indicates the appropriateness of the conducted KDE process. The degree of flatness in these uniform distributions serves as a key indicator of the accuracy of the KDE process. In the final stage, the inverse CDF of the standard Gaussian PDF is applied to all uniform distributions, as shown in the right-most figure. As illustrated in Fig. 6, a bivariate joint PDF is depicted for each uncertainty factor within a microgrid, along with the net load forecast error. The median of each dataset is represented by a zero value. A clear correlation is noticeable between wind forecasting and net load forecast error. When considering wind forecasting and PV forecasting, there is an increase in the absolute value of the net load forecast error as the forecast value rises. Conversely, there is a relatively weak correlation between load demand forecasting and net load forecast error. This result suggests that forecast values tend to be less accurate when high PV or wind generation is expected. When planning microgrid operations, a more conservative strategy is necessary during these conditions. In contrast, load demand forecast values show a weaker correlation with forecast errors. Thus, no specific action is required based on load demand forecast values.

**Figure 7:**
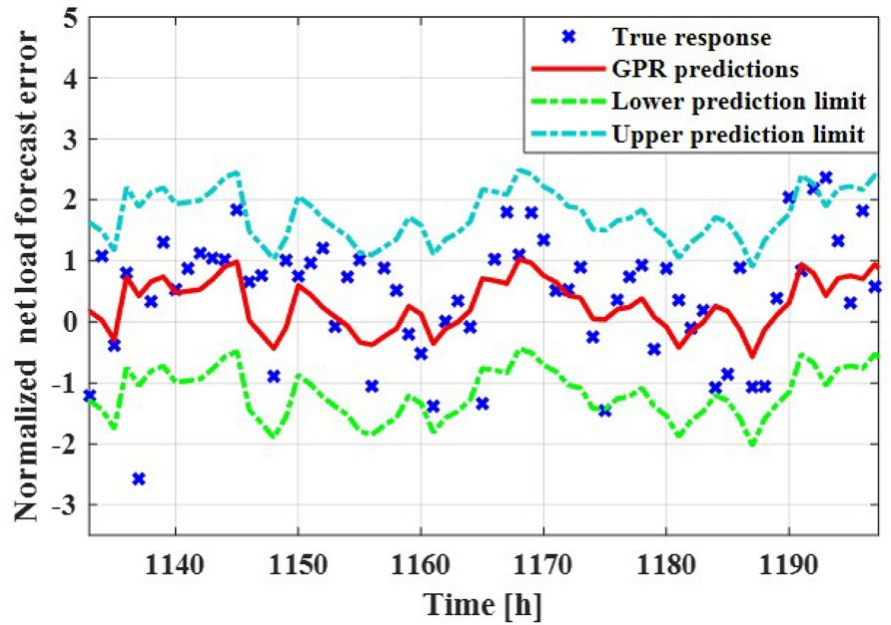
Comparison between actual net load forecast error values and GPR estimation.

Given the presence of PV generation spanning the entire day, two distinct covariance matrices are introduced. One for daytime and another for nighttime. The covariance matrices for each Gaussian copula model, corresponding to daytime and nighttime, are formulated as follows:

$$\begin{aligned} C_{day}= & {} \begin{bmatrix} 0.9594 &{} \quad 0.0484 &{} \quad 0.1159 &{} \quad 0.2950 \\ 0.0484 &{} \quad 0.6648 &{} \quad 0.095 &{} \quad 0.1333 \\ 0.1159 &{} \quad 0.0095 &{} \quad 0.9433 &{} \quad -0.0130 \\ 0.2950 &{} \quad 0.1333 &{} \quad -0.0130 &{} \quad 0.9569 \\ \end{bmatrix} \\ C_{night}= & {} \begin{bmatrix} 0.9590 &{} \quad 0.4969 &{} \quad 0.0748 \\ 0.4969 &{} \quad 0.9571 &{} \quad 0.1561 \\ 0.0748 &{} \quad 0.1561 &{} \quad 0.6937 \\ \end{bmatrix} \end{aligned}$$

Conditional PDF of net load forecast error can be calculated by using a covariance matrix and joint PDF.

**Figure 8:**
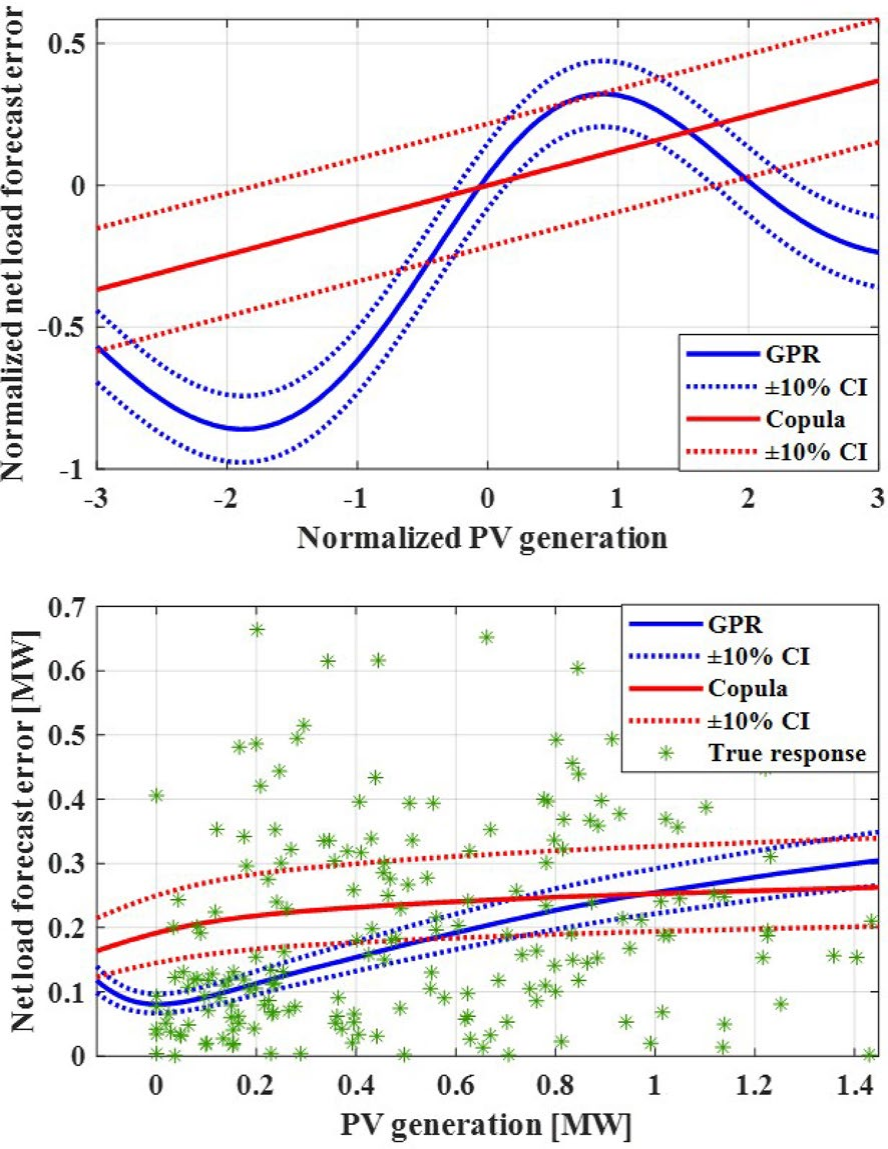
Comparison between actual net load forecast error values and GPR estimation as well as copula estimation.

The GPR model is constructed based on normalized uncertainty elements, namely PV, wind speed, and load demand. The kernel function outlined in Eq. (6) is employed for the GPR model. The optimal hyperparameter for the kernel function is determined as $\sigma _{f} = 0.88564$ to achieve the most accurate estimation. In Fig. 7, the GPR estimation of net load forecast error is presented for hourly point forecasts of uncertainty factors, along with its corresponding lower and upper limits delineated by a 90% Confidence Interval (CI). The visual representation highlights that the majority of actual net load forecast error values are dispersed within the two limits established by the GPR model.

The conditional PDF of the net load forecast error is illustrated in Fig. 8 concerning the forecasted PV generation. In this context, both wind generation forecast and load demand forecast are constrained within specific ranges (Wind = 5.3 ± 1 m/s, load = 1.95 ± 0.1 MW). Each conditional PDF, generated using copula and GPR models, is represented with a 10% CI.

In the upper panel of Fig. 8, it is evident that with an increase in the normalized value of PV generation, the copula-based estimation yields a linear growth of normalized net load forecast error, whereas the GPR-based forecast produces a non-linear curve. A comparison between the results of the two estimation models for net load forecast error and the actual net load error values (after reverting normalization) is depicted in the lower part of Fig. 8.

The adequacy of the conditional PDF can be evaluated through the assessment of the likelihood function value, as described by Myung and Pitt^26^. Likelihood function values enable the evaluation of the joint density at the observed data sample with respect to the provided parameters denoted as $\theta$. In this context, $\theta$ forms a vector encompassing the observed load, PV, and wind data.The expected likelihood value can be calculated as

11 $$\begin{aligned} {\hat{l}}(\theta ,x)=\prod _{i=1}^{n}\ln {f(x_i|\theta )}, \end{aligned}$$

where this expectation is taken with respect to the true density of observed net load forecast error *x*. The likelihood function values throughout the operational period are 0.3549 for the copula-based model and 0.397 for the GPR-based model. Consequently, the GPR-based model demonstrates superior fitness for predicting the actual net load forecast error in comparison to the copula-based model.

**Table 2:** Microgrid system description for case study.

	Copula-based forecast	GPR-based forecast
MLE value	0.3549	0.397

## Conclusion

This paper introduces a novel approach for quantifying stochastic net load forecast error within a microgrid system, considering the uncertainties associated with various elements. DERs like PV and wind generators are influenced by natural factors, while load demand is affected by seasonal changes and temperature fluctuations. Formulating a unified covariance matrix to model the aggregation of these diverse uncertainties is intricate, leading to the adoption of a GPR to capture intricate interrelations among them. To accommodate the dissimilar value ranges and unknown distributions of each uncertainty element, normalization techniques such as KDE and CDF are employed.

The PDF of net load forecast error is estimated using both GPR-based and copula-based models, followed by the validation of each model’s performance using real-world data. The study reveals that the GPR-based model outperforms the copula-based counterpart. Notably, the GPR-based model yields higher MLE values for the conditional PDF than those obtained from the copula-based model.

Given the insights garnered from the PDF analysis of net load forecast error, there is a need for further research to identify cost-effective optimal solutions for microgrid operation incorporating ESS and fuel cells. As the number of uncertainty elements grows, the complexity of interrelations among these elements will also increase, emphasizing the requirement for more in-depth investigation in future endeavours.

## Methods

All data generated or analysed during this study are included in this published article and its supplementary information files. Also, the datasets used during the current study available from the corresponding author on reasonable request.

### Supplementary Information

Supplementary Information.
